# Techno-economic and environmental analyses of the pyrolysis of food waste to produce bio-products

**DOI:** 10.1016/j.heliyon.2024.e27713

**Published:** 2024-03-13

**Authors:** Mohammad Alherbawi, Prakash Parthasarathy, Samar Elkhalifa, Tareq Al-Ansari, Gordon McKay

**Affiliations:** Division of Sustainable Development, College of Science and Engineering, Hamad Bin Khalifa University, Qatar Foundation, Doha, Qatar

**Keywords:** *Food waste*, *Pyrolysis*, *Empirical model*, *LCA*, *Bio-products*

## Abstract

Food waste has become a source of concern as it is generated abundantly worldwide and needs to be valorised into new products. In this study, cucumber, tomato, and carrot wastes were investigated as pyrolysis feedstocks as a single component (cucumber), a binary component mixture (cucumber and tomato), and a ternary component blend (cucumber, tomato, and carrot). Fourteen scenarios were simulated and evaluated based on varying the feedstock blend (single, binary, and tertiary), temperature (300 and 500 °C), and feedstock moisture content (5, 20, and 40%). Using an established empirical model, the effect of these parameters on product yields, techno-economic implications, energy requirements, and life cycle analysis (LCA) outcomes were investigated. The best performers of each scenario were determined, and their strengths and weaknesses were identified and compared with other scenarios. In terms of product yields, all three systems (single, binary, and tertiary) followed a similar pattern: bio-oil yields increased as temperature and feedstock moisture content increased, while biochar yields decreased as temperature and feedstock moisture content increased. The production of syngas, on the other hand, was only observed at elevated temperatures. The total energy requirement exhibited an increase with increasing temperature and feedstock moisture content. The economic evaluation revealed that the return on investment (ROI) value for the single component at 5% moisture content at 300 °C is 29%, with a payback period (PB) of only 3.4 years, which is potentially very appealing. The water footprint increased with increasing pyrolysis temperature but decreased with increasing moisture content in all scenarios. The land footprint is observed to remain constant despite changes in process conditions. The study's findings contribute to the pyrolysis process's scalability, technological advancement, and commercialisation.

## 1 Introduction

The food supply chain is characterised by significant losses and waste, from the earliest stages of agricultural production through to the final consumption of food by households. Since food waste accumulates in vast quantities, has negative environmental impacts, and is putrescible, many countries contend with this issue on a national level and conduct analyses based on the Water-Energy-Food (WEF) nexus models. Around 1.6 billion tonnes of palatable food are wasted annually worldwide, equal to one third of what is produced for human consumption [1,2].

Crop residues, catering waste, and mixed domestic food waste have all been classified as food waste. Food waste contains a variety of constituents, including proteins, carbohydrates, amino acids, lipids, vitamins, phosphates, and carbon ingredients, making it a favorable source for the production of chemicals and fuels [3]. However, the high moisture composition of food waste limits its valorisation. Traditional waste management techniques, such as composting, combustion, and anaerobic digestion, face an additional obstacle brought on by the salty nature of food waste. Recent research has proven that the pyrolysis process is an effective method for addressing the problems that have been outlined above [4].

Recent decades have seen increased attention to pyrolysis for the management of food waste due to its effectiveness in valorising biomass/carbon-rich materials into biofuels/bio-products. Pyrolysis is a thermochemical conversion process that involves heating feedstock to temperatures above 400 °C with little or no oxygen present [5,6]. It has a lower environmental impact than incineration [7]. The ability to handle all types of feedstocks and deliver varied bio-products (*e.g.,* biochars, pyro-oil (bio-oil), and pyrogas (syngas) is another advantage of using the pyrolysis process [8,9]. In addition, the method consumes less reaction time than biochemical processes, and it is also easily scalable. Specifically, in recent years, pyrolysis has been used to produce biochar for soil amendment applications such as carbon sequestration, and to provide food and water security in the world's hot and dry regions [10].

After potatoes, tomatoes are the world's second most popular vegetable, while cucumbers and carrots are two of the ten most popular veggies. According to the report by FAOSTAT, the world produces approximately 182 million tonnes of tomatoes, close to 75 million tonnes of cucumbers and gherkins, and nearly 40 million tonnes of carrots and turnips [11]. Because tomatoes, carrots, and cucumbers are highly consumed, enormous amounts of their waste are generated each year. The aforementioned vegetables, despite their rich moisture content, have a high volatile composition, making them prospective energy sources. As a result, tomatoes, carrots, and cucumbers are investigated as potential pyrolysis feedstocks in this study.

Techno-economic analyses (TEA) are commonly used to determine the economic viability of a process and its output. Life cycle assessment (LCA), on the other hand, is used to assess, integrate, and interpret the economic and social functioning of a process or product. The LCA aids in providing a clear picture of the decision-making process by identifying inputs and outputs, analysing energy and environmental impact scenarios, and taking cost-effectiveness into account, all of which have an impact on the entire production process. The trade-offs between environmental and economic performance can be effectively highlighted by combining TEA and LCA [12].

The use of the ASPEN plus (Advanced Process Engineering System) modeling software in process engineering applications has become more common in recent decades. The tool is widely applied to as it can be used for all phases of materials (solid, liquid, and vapour). According to recent research studies, the software can also be used to analyse pyrolysis studies and predict the composition of pyrolysis products [13]. In addition, it can be used to optimise pyrolysis operating conditions like temperature, solid residence time, heating rate, and feedstock size.

Researchers have so far developed four models to predict pyrolysis products and their compositions. They are kinetic models, empirical models, mechanistic models, and chemical equilibrium-based models [14]. In spite of the availability of the aforementioned models, empirical models are frequently used to forecast the distribution of pyrolysis products since they accurately predict their composition. Models based on empirical data are developed mathematically to explain and predict empirical results by mathematically optimising a set of reported experimental data. With regard to the pyrolysis process, pyrolysis reactions are used as the basis of the model. The model also covers pyrolysis regulating parameters such as heating rate and temperature.

The pyrolysis process is an energy-intensive process that uses elevated temperatures and includes a pre-drying stage to reduce the moisture content of the wet biomass sample. Furthermore, the feedstocks need size-reduction (grinding), which has energy requirements that must be identified and accounted for during the process. Thus, in this study, the LCA of food waste pyrolysis is performed from cradle to gate, evaluating the process's carbon, energy, land, and water footprints. Where the yield and composition of the product, as well as the energy requirement, are calculated using an empirical model. Fourteen scenarios with varying feedstock, blending ratios, and operating parameters were set and evaluated. Each scenario is assessed solely in terms of its impact on the desired product, with the final decision based on industrial scenarios.

Numerous research investigations have been conducted on the pyrolysis of various vegetable waste materials, including but not limited to potato peel [15], cauliflower [16], onion skin [17], garlic skin [17], and banana flower petal [18]. Nevertheless, there is a scarcity of literary investigations regarding the pyrolytic characteristics of vegetable waste materials, such as cucumber, tomato, and carrot [19]. Therefore, this study examines the pyrolytic characteristics of cucumber, tomato, and carrot. In order to produce bio-products from lignocellulosic biomass, such as food waste, a substantial amount of feedstock materials would be required. This necessitates the utilisation of biomass blending instead of relying solely on a single biomass feedstock [20]. Additionally, it should be noted that the composition and quality of pyrolysis products are greatly influenced by the feedstock blend [21]. Consequently, this study investigates the pyrolytic properties of vegetable waste blends consisting of single (cucumber), binary (cucumber + tomato), and ternary (cucumber + tomato + carrot) components.

The prediction of pyrolysis bio-products using empirical models has been conducted by different researchers through the utilisation of various models. In their study, Sharma et al. utilised an empirical model to make predictions regarding the output of biochar and the composition of syngas resulting from the pyrolysis process applied to wood biomass [22]. The applied model employed empirical data in order to resolve the relevant equations. Nevertheless, the predictive model failed to incorporate temperature as a factor in its estimation of biochar yield. In a separate investigation conducted by Neves et al., a mathematical model was created, incorporating elemental balances, empirical relations, and energy balances [23]. The majority of the existing models focus exclusively on the features of feedstock materials, neglecting the influence of pyrolysis parameters. However, a smaller subset of models consider both feedstock characteristics and pyrolysis parameters [24]. This study is among a limited number of investigations that simultaneously evaluate the qualities of the feedstock and the parameters of the pyrolysis process. Moreover, the utilisation of predictive models in economic analysis is unusual, which has been performed in this study.

The objective of the study is to.

i. investigate the pyrolytic characteristics of single (cucumber), binary (cucumber + tomato) and ternary (cucumber + tomato + carrot) vegetable waste blends and investigates the effect of temperature and feedstock moisture content on the pyrolysis bio-products using an empirical model. For this, fourteen scenarios were simulated using a pyroysis simulator;
ii. carry-out techno-economic and LCA assessments for the fourteen-pyrolysis operating scenarios;
iii. to determine which scenario performs the best in terms of process conditions and feedstocks, and to identify each scenario's strengths and weaknesses.

Empirical models were constructed by utilising the outcomes of proximate and ultimate analyses conducted on the feedstock samples, in conjunction with the pyrolysis operating parameters. The study also assessed the TEA of pyrolysis in the fourteen scenarios mentioned earlier, focusing on the production of bio-oil, biochar, and syngas. This evaluation considers factors such as initial capital investment, operational production costs, power consumption, and relevant data from existing literature. There isn't much published data on a prediction model that handles sensitivity and techno-economic assessments of this kind.

## 2 Materials and methods

Cucumber (*Cucumis sativus*), carrot (*Daucus carota* subsp. *Sativus*), and tomato (*Solanum lycopersicum*) were the three vegetables studied in this study. These pyrolysis feedstocks were investigated as a single component system (cucumber), a binary component mixture (cucumber and tomato), and a ternary component blend (cucumber, tomato, and carrot). The binary (cucumber-tomato) and ternary (cucumber-tomato-carrot) blends were developed by combining equal amounts of individual components (by weight). First, the vegetable samples were dried for 24 h in a hot-air oven set to 140 °C.

### 2.1 Materials characterisation

The dried samples were subjected to moisture analysis, proximate analysis, and elemental analysis. The procedure reported by Choi et al. was followed while determining the moisture content [25]. The proximate analysis testing in this study was conducted using the ASTM D7582-12 standard using the Discovery SDT 650 instrument manufactured by TA Instruments, located in New Castle, USA. The elemental analysis was performed in accordance with ASTM D 3176-8 using a CHN elemental analyser (EURO EA3000, Euro Vector, Italy).

### 2.2 Model development and description

The investigation looked at pyrolysis, and the prior pre-treatment processing stages, which are grinding and drying. The two primary approaches for assessing process effectiveness and performance of the components are environmental and economic analyses based on products quality and product distributions. The investigation was based on the use of three main type of feedstocks-single (cucumber), binary (cucumber and tomato) and finally a tertiary blend (cucumber, tomato, and carrot), three moisture levels (5, 20, 40%) and two pyrolysis temperatures (300 and 500 °C). The possible scenarios for the techno-economic study of the pyrolysis process is presented in Table 1.

**Table 1:** Scenarios for the techno-economic study of the pyrolysis process.

Scenario	Component	Moisture level (%)	Temperature (°C)
1.	Single (Cucumber)	5	300
2.	Single (Cucumber)	20	300
3.	Single (Cucumber)	40	300
4.	Single (Cucumber)	5	500
5.	Single (Cucumber)	20	500
6.	Single (Cucumber)	40	500
7.	Binary (Cucumber and tomato)	5	300
8.	Binary (Cucumber and tomato)	20	300
9.	Binary (Cucumber and tomato)	5	500
10.	Binary (Cucumber and tomato)	20	500
11.	Tertiary (Cucumber, tomato, and carrot)	5	300
12.	Tertiary (Cucumber, tomato, and carrot)	20	300
13.	Tertiary (Cucumber, tomato, and carrot)	5	500
14.	Tertiary (Cucumber, tomato, and carrot)	20	500

**Fig. 1:**
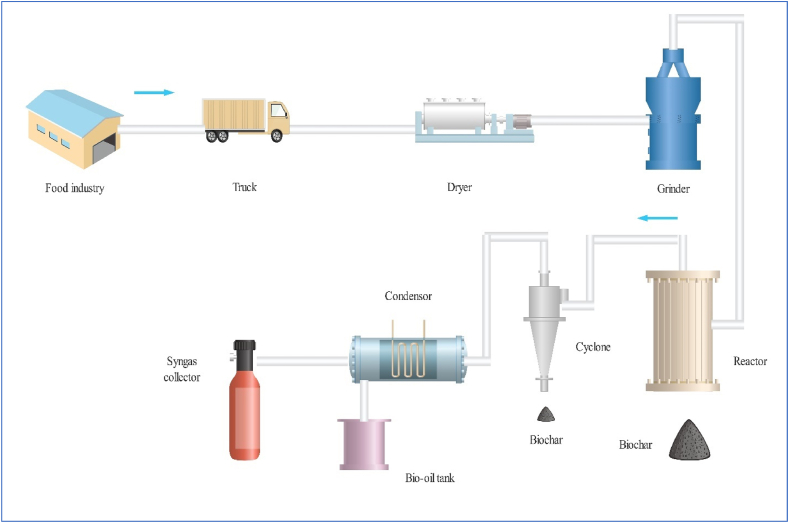
A streamlined flow diagram illustrating the pyrolysis of food waste.

The selected scenarios were based on varying the conditions of the process and the composition of the feedstocks. Process parameters included the process temperature, while the feedstock composition involved varying the moisture content of the feedstock in addition to the preliminary analyses (proximate and ultimate) of the feedstocks chosen for the TEA. The parameters explored in the analyses included product yield and properties, energy savings, environmental performance, and economic metrics. A pyrolysis process simulator was developed by utilising empirical equations reported by Abhijeet et al. [26] through the utilisation of a Microsoft Excel spreadsheet. The simulator primarily relied on the proximate and ultimate analyses of the feedstock, as well as the drying efficiency and pyrolysis temperature. Fig. 1 shows a simplified process flow diagram that illustrates how an empirical model was built for the pyrolysis degradation of the chosen food waste samples. Using an isothermal pyrolysis reactor with N_2_ as carrier gas, the feedstock samples were pyrolysed.

The prediction model was assumed to follow the below steps. Following the drying of samples, the feedstocks are thermally decomposed (pyrolysed) and transformed into H_2_O, bio-oil, biochar, and syngas (CO, CO_2_, CH_4_, and H_2_). Equations (1), (2), (3)) describe how these bio-product components are generated during the initial step of pyrolysis [27]. In the second step, further cracking of the bio-oil occurs with the rise in temperature. Syngas products generated during this phase are divided into gaseous components, depending on the biomass composition.

(1) $$Biochar\;yield\;in\;total=2.43*\exp\left(-0.66*T*10^{-2}\right)+0.106$$

(2) $$Bio-oil\;yield\;in\;total=Moisture\;composition\;of\;samples{+Y}_{bio-oil,F\;}+Y_{H_{2}O,F\;}$$

(3) $$Syngas\;yield\;in\;total=Y_{CO,F\;}{+Y_{CO_{2},F\;}+Y}_{H_{2,F}\;}+Y_{CH_{4},F\;}$$

The following Equations (4), (5), (6)) are used to determine the composition of carbon, hydrogen, and oxygen in biochar:

(4) $$Carbon\;content=0.93-0.92*\exp\left(-0.42*T*10^{-2}\right)$$

(5) $$Hydrogen\;content=(0.10*\exp\left(-0.24*T*10^{-2}\right)+\left(-0.41*10^{-2}\right)$$

(6) $$Oxygen\;content=0.85*\exp\left(-0.48*T*10^{-2}\right)+0.07$$

Neves et al. constructed an empirical model initially considering multiple reactors operating with a broad range of temperatures (i.e., 200–1000 °C) to replicate experiments using more than 60 biomass samples of varying nature [24]. In order to comprehend the pyrolytic behaviour, the obtained data were organised and analysed. By combining elements and characteristics of the products (bio-oil, syngas, biochar, and water), empirical relationships were derived from the biomass pyrolysis stoichiometry. The Cheng et al. model was used to estimate the energy needs of the system wherein Dulong's formula was used to determine the energy content of the products [28]. The current model is based on the aforementioned works and the assumed process conditions for the pyrolysis simulator is presented in Table 2.

**Table 2:** Assumed process conditions for the pyrolysis simulator.

Parameters	Values	Reference
Plant input capacity	20 T/h	
Operating temperature	300/500 °C	[29]
Drying efficiency (%)	60–95%	
Nitrogen flow rate	5 lit/min	
Reaction time	30 min	

### 2.3 Techno-economic evaluation

Cost estimation is a difficult task and this study investigates the impact of plant production capacity on minimum selling price (MSP), return on investment (ROI), and payback (PB) since these key factors have a significant implication on project economics. equations (7)–(19) that are employed in this study to estimate the economic parameters are presented in Table 3.

**Table 3:** Equations decsribing the economic parameters [30].

(7) CAPEX=∑Purchasedequipment+Equipmentsetting+Piping+Civil+Steel+Instrumentation+Electrical+Insulation+Paint+Contractfees+Generalandadministrativeoverheads+Contingencies
(8) $$Working\;capital=\frac{5\%\;of\;CAPEX}{lifetime}$$
(9) OPEX=∑Feedstocks+Operatingcharges+Labourcharges+maintenancecost+Plantoverhead+Generalandadmistrativeoverheads
(10) Subtotaloperatingexpenses=∑Operatingcharges+Labourcharges+maintenancecost+Plantoverhead
(11) Labourcharges=(OperatorspershiftXOperatorcharges)+(supervisorspershiftXSupervisorcharges)
(12) $$Operating\;charges=\frac{25\%\;of\;labour\;charges}{Period}$$
(13) $$Plant\;overhead=\frac{50\%\;of\;labour\;charges\;and\;maintenance}{Period}$$
(14) $$General\;and\;administrative\;cost=\frac{8\%\;of\;subtotal\;operating\;cost}{Period}$$
(15) $$Return\;on\;investment\;\left(\%\right)=\frac{Net\;profit}{CAPEX}$$
(16) $$Payback\;period\;\left(years\right)=\frac{CAPEX}{Cash\;inflow}$$
(17) $$Minimum\;selling\;price\;\left(\frac{USD}{kg}\right)=\frac{CAPEX+\sum_{1}^{lifespan}{(Opex\;\left(1+Discount\;Rate\right)}^{-lifespan})\;}{\sum_{1}^{lifespan}{(fuel\;yield\left(1+Discount\;Rate\right)}^{-lifespan})}$$
(18) $${Cost}_{design}={Cost}_{base}∙{\left(\frac{{Capacity}_{design}}{{Capacity}_{base}}\right)}^{scaling\;factor}∙Installing\;factor$$
(19) $${Cost}_{design,{\;USD}_{2019}}={Cost}_{design,{\;USD}_{i}}∙\left(\frac{{CEPCI}_{2019}}{{CEPCI}_{i}}\right)$$

For a plant hourly input capacity of 20 tonne, the economic feasibility of food waste pyrolysis is investigated. The plant is assumed to be functioning in Qatar. Below are the assumptions used in the economic analysis (Table 4.).

**Table 4:** Economic assessment assumptions.

Parameters	Values	Reference
Plant's location	State of Qatar	
Plant lifespan	25 years	[31]
Discount rate	20%	[31]
Annual operating hours	8000 h/y	[32]
Transportation distance	100 km	[29]
Analysis year	2019	
Vegetable waste procurement cost	75 USD/T	
N_2_ gas	0.15 USD/kg	[30].
Water	0.22 USD/m^3^	[30].
Electricity	0.07 USD/kwh	[30].
Biochar	0.2 USD/kg	[30].
Bio-oil	0.4 USD/kg	[29]
Syngas	0.056 USD/kg	[29]
Natural gas	2.545 USD/mmbtu	[29]
Ash disposal	16.3 USD/T	[29]
Wastewater discharge	0.27 USD/T	[29]

The pyrolysis equipment, accessories, and labour costs are derived from literature reported elsewhere. The Chemical Engineering Plant Cost Index (CEPCI) is a practical index that is used to account for changes in the value of goods and equipment over time and link those values to the performance of the global economy [33]. All costs are scaled up and inflated to the base year 2019 (Pre-Covid 19) using CEPCI.

### 2.4 Environmental lifecycle assessment

**Fig. 2:**
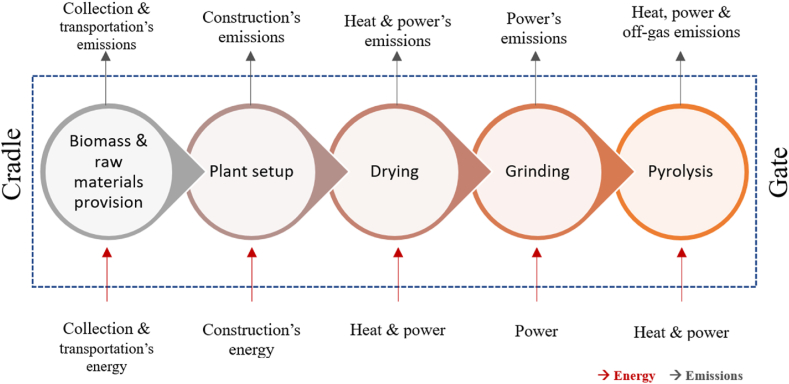
LCA scope of analysis for a food waste pyrolysis plant.

A cradle-to-gate LCA of food waste pyrolysis has been conducted. Two impact categories were selected: Global Warming Potential (GWP) in “tonne CO_2_-e” and energy footprint in “GJ.” All quantified inputs and outputs to the system were grouped and specified per year (Functional unit: one operational year). The overall scope of LCA is illustrated in Fig. 2.

At biomass collection stage, it was assumed that biomass is collected and transported over a distance of 100 km using diesel heavy trucks. The energy consumed and emissions released throughout this stage were adapted from literature as function of distance travelled and weight transported [34]. While the energy and emissions associated to nitrogen gas production were taken from literature reported elsewhere [35].

For the plant setup stage, the construction area required for the plant was estimated based on the refinery siting workbook as a function of plant's capacity [36]. Whereas the required energy and associated emissions per square meter were adapted from previous literature studies [37,38]. However, the embodied energy and emissions of equipment were neglected.

In addition, for the key pre-processing and processing stages, the energy footprint was estimated based on the findings in the previous modeling section, while the associated emissions were evaluated based on the sixth climate change report of the Intergovernmental Panel on Climate Change (IPCC). The GWP associated with utility usage and the greenhouse gases (GHG) released to atmosphere was evaluated using Equation (20) and Equation (21), respectively.

(20) $$CO_{2\;eq.}\left(utility\right)=E\times EF$$

(21) $$CO_{2\;eq.}\left(off-gas\right)={Mass}_{GHG}\times GWP$$

Where, *E* is the energy consumed, *EF* is the emission factor related to the utility, and *GWP* is the global warming potential value for each GHG components.

## 3 Results and discussion

Fundamental research into the physicochemical properties of vegetable wastes is essential for understanding the overall process and designing process parameters for product generation. For *e.g.* Moisture analysis aids in the design of handling, storage, drying, and feeding equipment and thermochemical conversion approaches, ash analysis aids in the estimation of potential clinkering, fouling, and slagging problems during pyrolysis [39], elemental analysis aids in the improvement of conversion efficiency, and heating value analysis aids in the enhancement of energy recovery efficiency. A TEA of vegetable waste pyrolysis is required to determine its commercial viability. After calculating the mass and energy balances, an economic analysis was performed to estimate the yield of pyrolysis products, the cost of pyrolysis product production, and the profit gain. Life cycle GHG emissions are the total of direct and indirect GHG emissions that occur during all stages of a product's life. LCA results provide a more detailed framework for evaluating waste management strategies, identifying environmental impacts and hotspots in the waste treatment hierarchy. The analysis assesses the environmental burdens and potential impacts of processes by compiling a list of inputs and outputs and interpreting the study's findings. The findings of the feedstock attributes, techno-economic, and LCA analyses have been discussed in this section.

### 3.1 Feedstock attributes

**Fig. 3:**
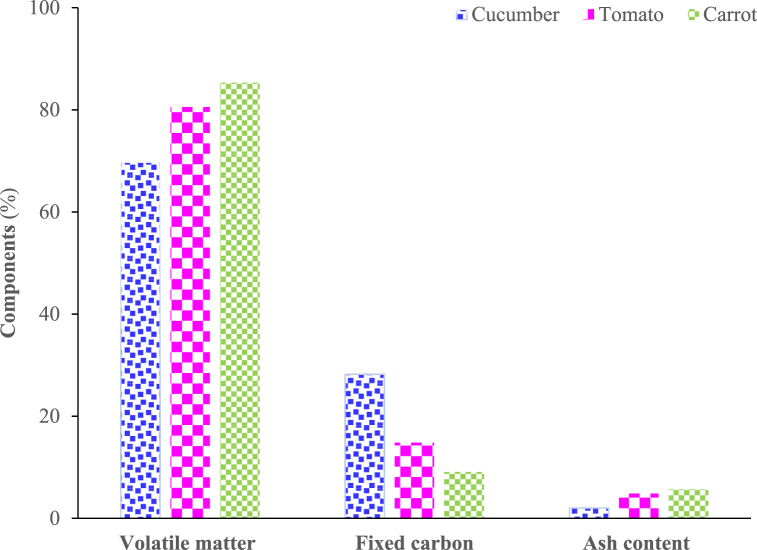
Proximate analysis results of vegetable wastes.

The vegetables displayed moisture contents of 96 wt%, 95 wt%, and 90 wt% for cucumber, tomato, and carrot, respectively. The proximate analyses were performed on a dry basis, with at least three runs performed in triplicate to obtain three consistent values within 5%. Fig. 3 depicts the results of the proximate analysis of the three vegetable food wastes.

**Fig. 4:**
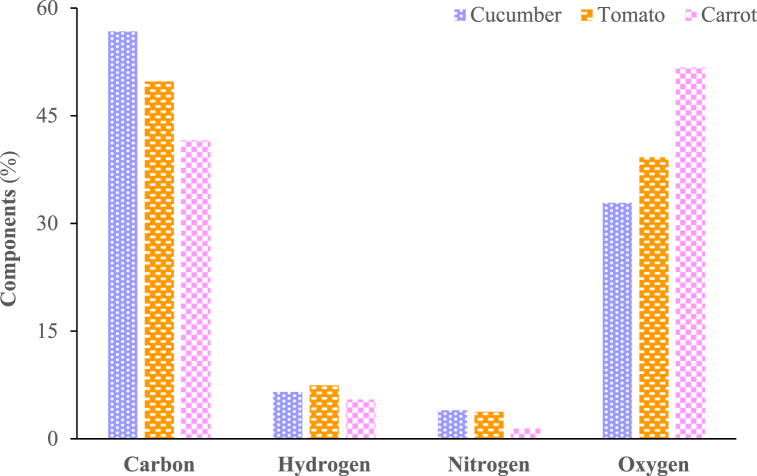
Ultimate analysis results of vegetable wastes.

The ultimate analysis runs were performed on a dry and ash-free basis. The values are based on the average of three measured results for each sample that are within 6% of one another. Fig. 4 depicts the results of the ultimate analysis of the three vegetables.

**Fig. 5:**
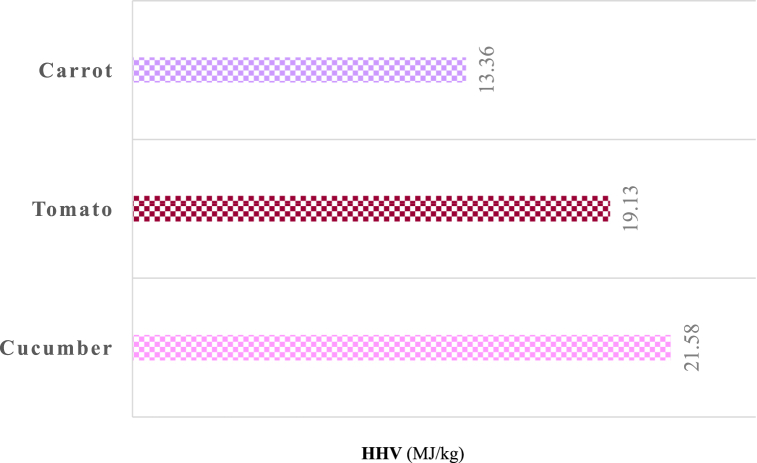
HHV analysis results of vegetable wastes.

The higher heating values (HHV) of the feedstock samples were calculated based on the elemental analysis results using the following correlation (Equation (22)) proposed by Channiwala and Parikh [40]. Fig. 5 shows the results of the HHV analysis of the vegetable samples.

(22) $$HHV\;\left(\frac{kJ}{kg}\right)=349.1\;C+1178.3\;H+100.5\;S-\;103.4\;O-\;15.1\;N\;–\;21.1\;Ash$$

### 3.2 Techno-economic analysis (TEA)

A TEA evaluates the economic viability of a process/product during its development phase in order to guide research, development, and investment [41]. The key issues of the TEA encompass the examination of the product's market worth, the selling price, and the investment expenses [42]. Moreover, it is essential to consider the values derived from forecasting future cash flows, as well as the anticipated ROI. The Pyrolysis Simulator was used in this study to generate economic parameters in addition to energy intensity of the different stages and the LCA.

#### 3.2.1 Technical analysis

For the first 3 scenarios, the synthesis of bio-oil and biochar decreased while moisture composition of the final product was high and syngas was not formed since it requires a higher production temperature. There are significant impacts on the yield and product quality associated with the high moisture content of the feed. The yields of the three products, syngas, bio-oil and biochar-for each temperature, have been compared with the system variables and are presented in Table 5, which compare the mixture blend of products – bio-oil, biochar, and syngas and for each simulation run.

**Table 5:** Pyrolysis products yields at different temperatures at different feedstock moisture content.

Biomass component	Feedstock moistute content (%)	Temperature (°C)	Scenario	Products yields (%)		
				Bio-oil	Biochar	Syngas
Single (Cucumber)	5	300	1	39	37	0
	20		2	33	31	0
	40		3	25	23	0
	5	500	4	55	18	5
	20		5	46	15	4
	40		6	34	11	3
Binary (Cucumber and tomato)	5	300	7	37	38	0
	20		8	31	32	0
	5	500	9	52	18	6
	20		10	44	12	5
Ternary (Cucumber, tomato, and carrot)	5	300	11	34	39	0
	20		12	28	33	0
	5	500	13	49	18	8
	20		14	40	18	8

For a cucumber feed moisture content of 5%, the bio-oil yield increases from 39% to 55% from 300 to 500 °C, while the biochar yield decreases from 37% at 300 °C to 18% at 500 °C. The production of syngas only occurs at the highest temperature of 500 °C with a 5% production level. Similar trends in product yield are observed at higher moisture contents of 20% and 40%, resembling the yield of feedstock with a moisture content of 5%. However, as the moisture content of the feed increases, the yields for each product decrease. This can be attributed to the higher moisture contents present in the products derived from the feed material. It can also be noted that the feedstock containing 40% moisture resulted in the lowest production of bio-oil and biochar at both temperatures (300 and 500 °C), contrary to the objective of achieving higher yields of bio-oil and biochar. Therefore, the case with a moisture level of 40% was not further pursued using binary and tertiary blends.

In the case of binary feedstocks, the trend in the products yields with temperature and moisture content are similar to those for the single component cucumber, that is, the bio-oil yields increase with increasing temperature and the biochar yields decrease with increasing temperature. Nevertheless, there exists a distinction in the generation of syngas, whereby the syngas production rates for the binary system at a temperature of 500 °C have exhibited an increment of around 1–2% compared to the corresponding values seen in single-component moisture content conditions.

The results of the ternary system, comprising cucumber, tomato and carrot, are shown in the table at the two temperatures and for the two same feed moisture contents, namely, 5% and 20%, as in the binary systems. The trend in the three products yields with temperature and moisture content again follow a similar pattern to those for the single and binary component systems - namely, the bio-oil yields increase with increasing temperature and the biochar yields decrease with increasing temperature. However, there is a difference in the production of the syngas similar to that observed in the binary system with the syngas. The syngas production levels of the ternary system at a temperature of 500 °C have exhibited an increase beyond the levels observed in the moisture level equivalent to binary component moisture content levels, by an additional 1–2%.

The table shows the biochar product yields at the two temperatures for single cucumber feed moisture contents of 5, 20 and 40%. The biochar yield decreases 37% at 300 °C to 18% biochar yield at 500 °C at 5% moisture and from 37 % yield at 300 °C (5% moisture) to 23% biochar at 300 °C (40 % moisture feed). The bio-oil yields at 5% feed moisture content increase from 39% to 55% as the temperature increases from 300 °C to 500 °C, respectively. The results indicate that the bio-oil yields decrease with increasing moisture content. At 5% moisture and 300 °C, the bio-oil yield is 39% but at 40% moisture feed, the oil yield has fallen to 25% at 500 °C. At 40% moisture content, the yield is 34% but still quite a bit lower than the 55% bio-oil yield at 500 °C at 5% moisture in the feed. The effect of temperature and feed moisture content on the syngas production yields are presented in the table. In fact, in the single component cucumber system syngas is only produced at 500 °C giving 3%, 4% and 5% syngas at 40, 20 and 5% feed moisture levels respectively.

A similar increasing and decreasing trend in the yield of bio-oil and biochar with the increase in temperature was noticed in tomato peel pyrolysis by Prasad and Murugavelh [43]. The researchers conducted pyrolysis experiments at varying temperatures between 450 and 600 °C. The bio-oil yield was found to be 14% at 450 °C, 18% at 500 °C, 32% at 550 °C, and 40% at 600 °C. The biochar yield was observed to be 44% at 450 °C, 39% at 500 °C, and 26% at 550 °C. However, at a temperature of 600 °C, a slight increase in the biochar yield (28%) was observed. Concurrently, the rise in temperature resulted in a varied reaction in the production of syngas. An increase in the yield of syngas, from 42% to 44%, was noticed when the temperature was raised from 450 to 500 °C. However, a decrease in syngas yield, from 44% to 32%, was detected when the temperature was further increased from 500 to 600 °C. Almutairi et al. [44] also noted a decline in biochar yield with increasing temperature in the pyrolysis of cucumber plant waste. The biochar yield at 300 °C, 400 °C, 500 °C, and 600 °C was recorded as 57%, 55%, 48%, and 44%, respectively. Pinto et al. [45] also observed that when pyrolysis temperature of carrot waste is increased, the yield of biochar decreased. The biochar yields at temperatures of 200 °C, 300 °C, 400 °C, 500 °C, and 600 °C were found to be 78%, 45%, 33%, 32%, and 31%, respectively.

For the binary cucumber-tomato system only two moisture contents were simulated, namely, 5% and 20%; the biochar yields decrease with increasing temperature and decrease with increasing feed moisture content. For the bio-oil production in the binary system, the trends in the yields followed a similar pattern to the single component systems – increasing with increasing temperature and decreasing with increasing feed moisture content. In the case of the syngas yields an interesting phenomenon can be observed in this binary system, namely, that for both moisture contents, syngas was produced at the highest temperature 500 °C producing a different result from the syngas production in the single component simulations. This could be due to blending synergy or the lower total carbon content in the binary feed blend.

The next system that was studied is the ternary system of cucumber-tomato-carrot and the results are presented in the table which shows the biochar yields at 5% and 20% moisture contents at 300 °C and 500 °C and the yield are decreasing with increasing temperature and with increasing moisture content. It is interesting to compare the biochar yields for the single component, the binary system and the ternary system at 5% moisture and 300 °C produces biochar yields of 37%, 38% and 39% respectively and these correspond to initial feed carbon contents of 59%, 55% and 51% respectively. So, although the results are very close they are not following the carbon content pathway and there may be some synergy by creating blends of food waste materials. The bio-oil yields increase with temperature and decrease with moisture content. A similar comparison can be performed for the single, binary and ternary component systems at 300 °C and 5% moisture content the oil yields are 39%, 37% and 34% respectively, Hence, the bio-oil fraction is decreasing as the system becomes more multicomponent. The syngas yields for the ternary system are presented in the table and show very interesting results. Similar, as in the binary system, syngas is produced at the highest temperature 500 °C and in greater yields than in the binary system under equivalent conditions.

It is apparent from the effect of temperature on the pyrolysis results of the biochar yields that all biochar yields decrease with increasing temperature. This is to be expected, as more volatiles are driven off the feed materials with increasing temperature and the carbon content of the char increases due to the stable fixed carbon content. The increasing bio-oil yields with increasing temperature are a consequence of the increased quantity of volatiles emitted with increasing temperature in the range of 300 °C–500 °C, prior to the rapidly increasing production of syngas at temperatures above 500 °C. In the cucumber-tomato binary system and the ternary system, for both moisture contents, syngas was produced at 500 °C producing a different result from the syngas production in the single component simulations. This could be due to blending synergy or the lower total carbon content in the binary and ternary feed blends.

#### 3.2.2 Energy requirements

**Fig. 6:**
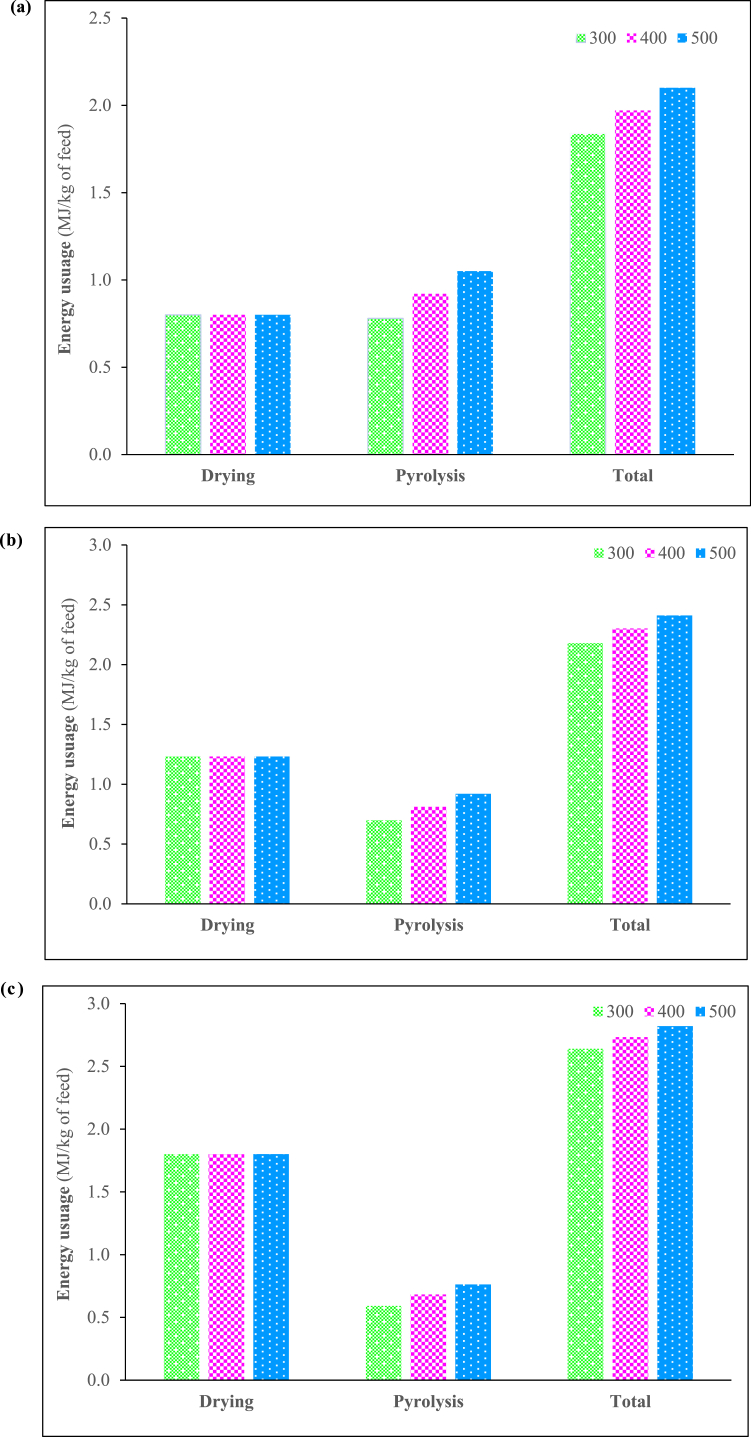
**(a.)** 5% Feedtsock moisture content, **(b.)** 20% Feedtsock moisture content, **(c.)** 40% Feedtsock moisture content.

Energy requirements were observed to be significantly affected by the high moisture content of the feedstock [46], putting more weight on the drying stage which for a 40% moisture content requires an additional 56% of energy when compared to the feedstock with 5% moisture content effectively doubling the energy requirement. The energy requirements of grinding remained unchanged while that of pyrolysis exhibited a slight change. The energy requirements for 5% feedtsock moisture content, 20% feedtsock moisture content, and 40% feedtsock moisture content are summarised in Fig. 6a and. b, and Fig. 6c respectively.

The total energy requirements for the systems for the production of the three products, syngas, bio-oil, and biochar, have been compared with the system variables and are presented in the figure. The total energy requirement increases with increasing temperature from 1.84 MJ/kg feed at 300 °C to 2.10 MJ/kg feed at 500 °C for a 5% moisture content and the energy requirement also increases with increasing moisture content – at 5% and 300 °C the total energy requirement is 1.84 MJ/kg feed and at 40% moisture and 300 °C the total energy requirement is 2.64 MJ/kg feed. Since energy cost is a major factor in the techno-economic assessment of a project, the detailed contributions of the energy requirements were subdivided into the drying component and the pyrolysis component.

The figures show the energy required for drying one kg of feed at 5% moisture content is 0.80 MJ/kg feed and is fairly constant at a pyrolysis temperature of 300 and 500 °C, because the moisture is driven off well below the pyrolysis temperature usually below 200 °C. However, by inspection of the drying energy requirement at the higher moisture contents of 20% and 40% the energy usage is increased. From the baseline level of 5% moisture to 20% and 40% moisture, the energy requirement increases by 54% and 125% respectively. This reflects many literature sources reporting that wet food waste is unsuitable for application to biochar pyrolysis and gasification because of the extensive energy requirements and therefore the excessive costs. In fact, at 20% and 40% feed moisture content, the energy contribution for drying is 56% and 68% respectively of the total energy requirement. These data provided the justification for the initial drying studies in this research project to find a cheap source of low value or waste energy that could provide the drying of the wet food wastes prior to pyrolysis. The opportunity to use flue gases in the temperature range 120–180 °C was the basis of this initial drying study. The results of this initial study demonstrated that drying of food waste could be achieved at these temperatures, but the drying time was a crucial factor to dry the food wastes down to a 5%–10% moisture content. This low dryness level could only be achieved using the elevated temperatures of 160 °C and 180 °C within 2 or 3 h in the initial experimental drying studies. The prolongation of lower temperatures would lead to a significant increase in the duration of the drying pre-treatment step, thereby causing it to become the limiting step in the overall pyrolysis process. Consequently, a larger volume of hold-up dryers would be necessary to fulfil the drying requirement within an adequate time limit. As a result, larger equipment would be required, resulting in higher capital expenses. Nevertheless, the use of the flue gas at 180 °C for pre-drying can meet the drying requirements and would be a major asset to the application of wet food waste in pyrolysis.

The energy for the pyrolysis processes itself is only shown in the figures for the two temperatures and at the three feed moisture contents of 5, 20 and 40%. For all three moisture contents, the trend with increasing temperature follows the same pattern, that is, the energy requirement per kg of feed increases. This is expected since, as temperature increases, more volatiles are driven off during pyrolysis, therefore consuming more energy per kg feed. As the moisture content increases, the pyrolysis energy values decrease based on pyrolysing one kg of wet feed because the amount of solid food waste (the fraction to be pyrolysed) in one kg of wet feed is decreasing with increasing moisture content.

The energy requirement values for the binary and ternary systems are not shown as they are the same values as for the single components, having the same solid waste food fractions and the same moisture contents.

#### 3.2.3 Economic performance

**Fig. 7a:**
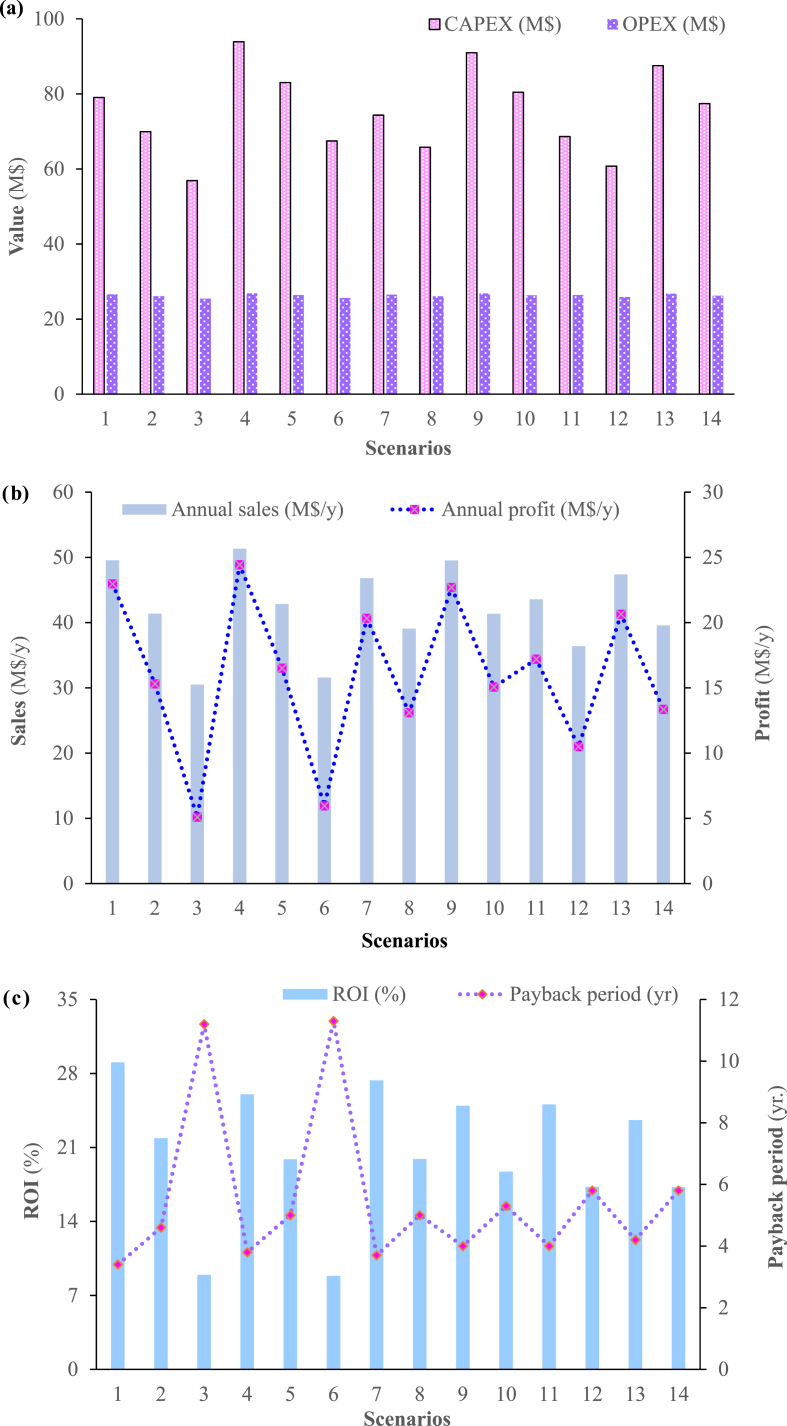
CAPEX and OPEX; **7b.** Annual sales and Annual profit; **7c.** ROI and PB period.

Full details of the economic analyses are presented in the project files in terms of capital expenditure (CAPEX), operating expenditure (OPEX), sales revenue, and return on capital investment (ROI). Since the ROI provides a major indicator of the feasibility of a project these values have been summarised. The economic parameters CAPEX and OPEX; Annual sales and Annual profit; and ROI and PB period are illustrated in Fig. 7a, **7b**, and **7c** respectively.

The ROI values are greater than 25% and suggest this project to produce biochar at 300 °C at 5% feedstock moisture content (at 29% ROI) but also 500 °C (at 26+%) provide potentially incredibly attractive opportunities and these projects have a PB in less than 4 years. The differences in the values can be attributed to different product distributions at different temperatures and the differences in the CAPEX and OPEX values, again due to operating at the higher temperatures. Even the ROI values for the 20% moisture feeds are quite positive with ROIs of the order of 20% and PB periods of about 5 years. At the higher moisture levels, the results are not so attractive with the ROIs falling below the 10% level and the PB period rising to over ten years.

Sales and profitability are the highest for the first scenario. This is due to the higher quality attributes of the final products (less moisture content of the final products and higher biochar and bio-oil yield). In addition, the ROI is the highest for scenario 1, making it the most favorable scenario thus far. Moreover, the PB period is reported to be the highest for the third scenario, which is the least favorable in terms of economics, product condition, and energy intensity.

For cucumber processed at a temperature of 500 °C at the three selected moisture contents of the feedstock, the increase in process temperature propelled the formation of syngas which decreased in quantity as moisture content increased. At the higher temperature, high bio-oil is formed in comparison with that found at a temperature of 300 °C. The quality of the final products decreases with increasing the moisture content of the feed which caused an increase in the moisture content of the final product.

Similar to the observations made for the first three scenarios, drying required more energy as moisture content increased, which can be translated into another 56% increase in requirements. The net energy requirements increased in comparison to scenarios involving less moisture content, with an increase witnessed for pyrolysis as well. In terms of sales and profitability, scenario 4 is the most favorable with an ROI of 26.02%, and the highest reported profit and sales, and shortest PB period. Scenario 4 is also the best in terms of energy savings and products attributes when compared with scenario 5, and scenario 6.

In scenarios 7 and 8, the analysis looks at the effect of increasing the moisture content while fixing the operating temperature for the cucumber and tomato feedstock mixture. It is observed that the exact same energy requirements of the two scenarios are shared with the first two scenario involving cucumber only. However, the production of biochar and bio-oil decreased. Furthermore, sales and profitability reduced in comparison to the first two scenarios for cucumber, with a decrease in ROI and an increase in PB period for the same temperature and moisture content.

For scenarios 9 and 10, it is proven once again that high temperatures promote the formation of syngas, which has a yield that is impacted by the moisture content of the feedstock. In comparison to scenarios 4 and 5, it is observed that the energy requirement are exactly the same. When compared to scenarios 7 and 8, sales and profitability decreased, ROI decreased, and PB increased. Hence, from the second type of feedstock type, scenario 7 is the most favorable.

In scenarios 11 and 12, the tertiary blend is considered for a processing temperature of 300 °C, while varying the moisture content of the feed. The biochar yield is the highest thus far for both scenarios. In comparison with scenarios 7 and 8, the energy intensity is the same. In agreement with the previous results, increasing the moisture content of the feed decreases both sales and profitability. Sales and profitability are lower than those reported for scenarios 7 and 8 when fixing the same moisture content for the feed. ROI has also decreased with the increase in moisture content of the feed. The PB increased when compared to scenarios 7 and 8, and when considering the impact of the feedstock composition.

For the last two scenarios, that is 13 and 14, interesting findings were observed. First, significant decrease in biochar yield and increase in bio-oil yields were observed with increasing the pyrolysis temperature. The net energy requirements are similar in magnitude to scenarios 9 and 10. Sales and profitability decrease with increasing moisture content in the feed. Here, the economic parameters are very similar to scenarios 11 and 12 with slight changes. This is reflected in the ROI which is almost the same for scenarios 11 and 13, and 12 and 14. Even the PB period is the same, when fixing the moisture content and looking at the two temperatures. The sales and profitability improved for scenarios 13 and 14 when compared to scenarios 11 and 12. The best scenario from the last 4 scenarios is 11 as it has the least moisture content, high economic value, and is associated with less energy requirements.

### 3.3 LCA results

The LCA involved studying the 14 scenarios in terms of their footprints. The break-up of contributions of each scenario is presented in Table 6. The system boundaries in LCA include: biomass collection, transportation, grinding, drying, pyrolysis and production of biochar and bioenergy (*e.g.* natural gas, electricity, fertilizer constituents like nitrogen), and application to soils in field [47].

**Table 6:** Summary of the LCA results from the Pyrolysis Simulator.

Scenario	Carbon footprint (ton CO_2_e/y)	Energy footprint (GJ/y)	Water footprint (m^3^/y)	Land footprint (ha/lifespan)
1	53,603	382,660	145,766	8.1
2	66,203	451,658	121,753	8.1
3	83,004	543,655	89,736	8.1
4	71,065	435,160	187,536	8.1
5	80,973	496,658	156,638	8.1
6	94,184	578,655	115,440	8.1
7	57,052	382,660	133,195	8.1
8	69,084	451,658	111,254	8.1
9	72,156	435,160	179,093	8.1
10	81,884	496,658	149,587	8.1
11	60,796	382,660	118,407	8.1
12	72,211	451,658	98,904	8.1
13	75,290	435,160	169,232	8.1
14	84,501	496,658	141,351	8.1

As can be deduced from the results in Table 6, and taking cucumber as the main feedstock, increasing the pyrolysis temperature is associated with an increase in energy demands and hence an increase in GHG emitted is observed. Similarly, increasing the moisture content of the feedstock requires additional processing especially in the grinding and drying stages, and thus require additional energy and as such additional GHG are released into the environment. Comparing the second type of feedstock, that is cucumber and tomato mixture, with the cucumber GHG results, it can be observed that the results did not exhibit a significant change, making them in the same magnitude regardless of the different composition of the new feedstock. It can be then concluded that the trend in values is the same for the first two feedstocks. Lastly, the third type of feedstock, that is the tertiary mixture, also exhibited an increase in GHG emissions with the increase in both temperature and moisture content. The increase is still in the same magnitude as the other types of feedstocks; however, it was the highest in GHG generation. Nevertheless, cucumber with moisture content of 40% and pyrolysis temperature of 500 °C resulted in the highest GHG emissions; even higher than those observed at 500 °C for the two other feedstocks. The similarity is attributed to the fact that the need for energy from natural gas and the use of electricity resulting from the energy requirements of the process are increasing, hence the trend is expected. The explanation for the similarity in the results is due to the drying stage, which brings the feedstock mixture to a certain extent of dryness preparing them for the pyrolysis stage at pre-determined conditions. Feedstock type and selection are vital when it comes to GHG emissions and production costs [48].

Energy is used and lost throughout the production stages of the biofuels. Hence, it is essential to account for the energy footprint. The results indicate that the feedstock type does not have any impact on the energy footprint, however increasing the pyrolysis temperature and similarly the moisture content increase their footprint. Again, it is evident that the impact of moisture content on the process and production energy footprint is significant as the 40% scenario for cucumber is linked to the highest energy footprint.

Water footprint is associated with the direct and indirect uses of freshwater in producing products and services [49]. Here, it is observed that water footprint increases with increasing the pyrolysis temperature but decreases with increasing moisture content for the same fixed type of feedstock. The trend is observed for the 14 scenarios. Lastly, the amount of land use, that is associated with the production of biofuel, or what is known as the land footprint is observed to stay the same regardless of the changes in process conditions, production scenarios, and energy requirements. This could be due to the fact that the land requirements for a given plant capacity is fixed.

## 4 Practical implications

The selection of the best alternative route should involve a trade-off of multiple criteria, while keeping the economics in line with the LCA results. The best route is not always the best route for the application, which means that the main application may involve a feedstock that is a blend of waste materials rather than a pure single waste feedstock when the pure performs best in the analyses. The goal of this study is to identify the best performers, identify their flaws, and optimise accordingly. If, for example, it is noted that biochar, which is better produced at moderate temperatures, is produced via a route that is found to be the least economical in comparison to routes favouring other products, then this is a choice that needs to be optimised.

According to the sensitivity analysis of the economic simulation results, increasing plant capacity results in an increase in ROI and a decrease in PB period. The impact of annual operating hours was observed to follow the same pattern as plant capacity; increasing the hours results in a reduction in PB periods while increasing ROI. Furthermore, increases in the market value of biochar (the product of interest in this study) increases ROI and shorten the PB period. The effect of moisture content is severe, so it was studied as well, and the results show that increasing the moisture content of the feed leads to a decrease in ROI and an increase in PB period.

The energy of biochar is also significant. Consider the following scenario: a transportation truck with a capacity of 5000 kg transports biochar. To calculate the volume share of biochar, a bulk density of 600 kg/m^3^ has been assumed. The volume share of biochar was worked out to be 8.33 m^3^. Based on LCA and energy footprint, the energy share of biochar alone was around 0.984 MJ/kg biochar for cucumber at 300 °C and moisture content of 5%. The figures are enormous, indicating that a significant amount of energy is expended in production. The fuel energy required for transportation must be calculated and considered when calculating the volume per energy share for biochar. This necessitates data input and scenarios that account for the distance between the manufacturing site and the receiving units.

Furthermore, and for practical purposes, the economic and LCA results must be aligned with other factors affecting the production of biochar and other products, such as long-term safety and environmental effects from plant operation [50]. This thorough analysis will aid in selecting the most appropriate scenario among the other alternatives and will ensure that the candidates chosen for optimisation at a later stage will meet the customer's demand. The process is guided not only by the use of software and the outputs provided, but it is also critical to consider industrial experience before making final decisions. Regarding the simulation results, it is important to note the negative sign for the syngas yield, which indicates that syngas production is hampered under the given conditions and that process conditions must be slightly altered to enable syngas production as well.

## 5 Conclusion

Food waste is a problem since it is produced in copious quantities worldwide and needs to be turned into new products. In this research, the pyrolysis feedstocks of cucumber, tomato, and carrot wastes were examined as a single component (cucumber), a binary component mixture (cucumber and tomato), and a ternary component blend (cucumber, tomato, and carrot). Based on modifying the feedstock blend (single, binary, and tertiary), temperature (300 and 500 °C), and feedstock moisture content (5, 20, and 40%), fourteen scenarios were simulated and analysed. The impact of these characteristics on product yields, techno-economic ramifications, energy requirements, and life cycle analysis (LCA) results was examined using a well-established empirical model. The top performers in each scenario were chosen, and their advantages and disadvantages were noted and contrasted with those in other scenarios.

All three systems (single, binary, and tertiary) produced bio-products with similar yields: bio-oil yields increased with temperature while it decreased with increasing feedstock moisture content, but biochar yields decreased with temperature and feedstock moisture content. On the other hand, syngas generation was only noticed at extremely elevated temperatures. The high moisture content of the feedstock was shown to have a substantial impact on energy consumption, which increased the pressure on the drying step. As the temperature and moisture content of the feedstock increased, so did the overall energy requirement. This is expected as, as temperature increases, the more volatiles are released during pyrolysis, hence costing more energy per kilogram feed.

The economic study found that the return on investment (ROI) value for the single component at 5% moisture content at 300 °C is 29%, with a payback (PB) period of only 3.4 years, which is potentially extremely enticing. Additionally, in this case, sales and profitability are high. According to LCA, increasing the temperature of pyrolysis is linked to higher energy requirements, which results in more greenhouse gases (GHG) being released into the atmosphere. Similarly, increasing the moisture content of the feedstock demands additional processing especially in the grinding and drying steps, and so require additional energy and as such additional GHG are emitted into the atmosphere. In both instances, the water footprint increased as the pyrolysis temperature increased but dropped when the moisture content increased. This can occur as a result of the same area of land needed for a certain plant capacity.

Detailed research will help choose the scenario that best fits the situation among the available options and guarantee that the candidates picked for optimisation at a later time will satisfy the needs of the client. Before making any final judgments, it is essential to reference industrial experience in addition to the software used and the outputs produced. The results of the study contribute to the scalability, technological development, and commercialisation of the pyrolysis process. The upcoming research should slightly modify the process parameters enabling syngas generation as well, since syngas production is hindered under the current circumstances.

## Funding

The team are especially grateful to the Qatar National Research Fund (10.13039/100008982QNRF) for the provision of an award under NPRP11S-0117–180328. Any opinions, findings and conclusions, or recommendations expressed in this material are those of the author(s) and do not necessarily reflect the views of QNRF or Qatar Foundation (QF). The team also extend their gratitude to the Qatar Environment and Energy Research Institute (QEERI) core analytical laboratory facilities for the provision of their analytical support.

## Code availability

Not applicable.

## Availability of data and material

Not applicable.

## CRediT authorship contribution statement

**Mohammad Alherbawi:** Writing – original draft, Validation, Methodology, Investigation, Formal analysis, Data curation. **Prakash Parthasarathy:** Writing – original draft, Validation, Methodology, Investigation, Formal analysis, Data curation, Conceptualization. **Samar Elkhalifa:** Writing – original draft, Methodology, Investigation, Formal analysis, Data curation. **Tareq Al-Ansari:** Writing – review & editing, Supervision, Resources, Project administration, Funding acquisition. **Gordon McKay:** Writing – review & editing, Validation, Supervision, Resources, Project administration, Methodology, Investigation, Funding acquisition, Conceptualization.

## Declaration of competing interest

The authors declare that they have no known competing financial interests or personal relationships that could have appeared to influence the work reported in this paper.

## Nomenclature

ASPEN: Advanced Process Engineering System
ASTM: American Society for Testing and Materials
CAPEX: Capital Expenditure
CEPCI: Chemical Engineering Plant Cost Index
CHN: Carbon Hydrogen Nitrogen
WEF: Water-Energy-Food
FAOSTAT: Food and Agriculture Organization Corporate Statistical Database
GHG: Greenhouse Gas
GWP: Global Warming Potential
HHV: Higher Heating Value
IPCC: Intergovernmental Panel on Climate Change
LCA: Life Cycle Assessment
MSP: Minimum Selling Price
OPEX: Operating Expenditure
PB: Payback
ROI: Return on Investment
TEA: Techno-economic analysis
USD: United States Dollar
