# Supplementary material for: Techno-economic and environmental analyses of the pyrolysis of food waste to produce bio-products
Source: Heliyon. 2024 Mar 13;10(6):e27713. doi: 10.1016/j.heliyon.2024.e27713 (PMC10958366; doi:10.1016/j.heliyon.2024.e27713)
Supplement: Multimedia component 1 [file mmc1.docx]

**Supplementary tables**

**Table 1.** Equations decsribing the economic parameters.

| **Equations** |  |
| --- | --- |
| $CAPEX=\sum Purchased equipment+Equipment setting+Piping+Civil+Steel+Instrumentation+Electrical+Insulation+Paint+Contract fees+General and administrative overheads+Contingencies$ | (7) |
| $Working capital= \frac{5\% of CAPEX}{lifetime}$ | (8) |
| $OPEX= \sum Feedstocks +Operating charges+Labor charges+maintenance cost+Plant overhead+General and admistrative overheads$ | (9) |
| $Subtotal operating expenses= \sum Operating charges+Labor charges+maintenance cost+Plant overhead$ | (10) |
| $Labor charges=\left( Operators per shift X Operator charges \right)+ (supervisors per shift X Supervisor charges)$ | (11) |
| $Operating charges= \frac{25\% of labor charges}{Period}$ | (12) |
| $Plant overhead= \frac{50\% of labour charges and maintenance}{Period}$ | (13) |
| $General and administrative cost = \frac{8\% of subtotal operating cost}{Period}$ | (14) |
| $Return on investment \left( \% \right)= \frac{Net profit}{CAPEX}$ | (15) |
| $Payback period \left( years \right)= \frac{CAPEX}{Cash inflow}$ | (16) |
| $Minimum selling price \left( \frac{USD}{kg} \right)= \frac{CAPEX+\sum_{1}^{lifespan} {(Opex \left( 1+Discount Rate \right)}^{-lifespan})}{\sum_{1}^{lifespan} {(fuel yield\left( 1+Discount Rate \right)}^{-lifespan})}$ | (17) |
| ${Cost}_{design}={Cost}_{base}\cdot\left( \frac{{Capacity}_{design}}{{Capacity}_{base}} \right)^{scaling factor}\cdot Installing factor$ | (18) |
| ${Cost}_{design,{USD}_{2019}}={Cost}_{design, {USD}_{i}}\cdot\left( \frac{{CEPCI}_{2019}}{{CEPCI}_{i}} \right)$ | (19) |

**Techno-economic results**

| **Scenario** | **Product Distribution** | **Net Energy Requirements** | **Economic Parameters** |
| --- | --- | --- | --- |
|  |  |  | - CAPEX (M$): 79.06 - OPEX (M$/y): 26.57 - Annual Sales (M$/y): 49.54 - Annual Profit (M$/y): 22.97 - ROI: 29.05% - Payback period (years): 3.4 |
|  |  |  | - CAPEX (M$): 69.95 - OPEX (M$/y): 26.07 - Annual Sales (M$/y): 41.37 - Annual Profit (M$/y): 15.30 - ROI: 21.87% - Payback period (years): 4.6 |
|  |  |  | - CAPEX (M$): 56.90 - OPEX (M$/y): 25.40 - Annual Sales (M$/y): 30.48 - Annual Profit (M$/y): 5.09 - ROI: 8.94% - Payback period (years): 11.2 |
|  |  |  | - CAPEX (M$): 93.90 - OPEX (M$/y): 26.88 - Annual Sales (M$/y): 51.31 - Annual Profit (M$/y): 24.43 - ROI: 26.02% - Payback period (years): 3.8 |
|  |  |  | - CAPEX (M$):83.03 - OPEX (M$/y): 26.35 - Annual Sales (M$/y): 42.86 - Annual Profit (M$/y): 16.51 - ROI: 19.88% - Payback period (years): 5.0 |
|  |  |  | - CAPEX (M$): 67.47 - OPEX (M$/y): 25.62 - Annual Sales (M$/y): 31.58 - Annual Profit (M$/y): 5.96 - ROI: 8.84% - Payback period (years): 11.3 |
|  |  |  | - CAPEX (M$): 74.35 - OPEX (M$/y): 26.47 - Annual Sales (M$/y): 46.80 - Annual Profit (M$/y): 20.33 - ROI: 27.34% - Payback period (years): 3.7 |
|  |  |  | - CAPEX (M$): 65.80 - OPEX (M$/y): 25.99 - Annual Sales (M$/y): 39.08 - Annual Profit (M$/y): 13.09 - ROI: 19.90% - Payback period (years): 5.0 |
|  |  |  | - CAPEX (M$): 90.98 - OPEX (M$/y): 26.82 - Annual Sales (M$/y): 49.51 - Annual Profit (M$/y): 22.69 - ROI: 24.94% - Payback period (years): 4.0 |
|  |  |  | - CAPEX (M$): 80.46 - OPEX (M$/y): 26.29 - Annual Sales (M$/y): 41.35 - Annual Profit (M$/y): 15.06 - ROI: 18.71% - Payback period (years): 5.3 |
|  |  |  | - CAPEX (M$): 68.64 - OPEX (M$/y): 26.35 - Annual Sales (M$/y): 43.56 - Annual Profit (M$/y): 17.20 - ROI: 25.06% - Payback period (years): 4.0 |
|  |  |  | - CAPEX (M$): 60.76 - OPEX (M$/y): 25.89 - Annual Sales (M$/y): 36.38 - Annual Profit (M$/y): 10.49 - ROI: 17.27% - Payback period (years): 5.8 |
|  |  |  | - CAPEX (M$): 87.53 - OPEX (M$/y): 26.75 - Annual Sales (M$/y): 47.39 - Annual Profit (M$/y): 20.64 - ROI: 23.58% - Payback period (years): 4.2 |
|  |  |  | - CAPEX (M$): 77.42 - OPEX (M$/y): 26.23 - Annual Sales (M$/y): 39.58 - Annual Profit (M$/y): 13.35 - ROI: 17.24% - Payback period (years): 5.8 |
